# IL-10 from dendritic cells but not from T regulatory cells protects against cisplatin-induced nephrotoxicity

**DOI:** 10.1371/journal.pone.0238816

**Published:** 2020-09-08

**Authors:** Wei Wei Wang, Yamei Wang, Kang Li, Raghu Tadagavadi, William E. Friedrichs, Madhusudhan Budatha, W. Brian Reeves

**Affiliations:** 1 Division of Nephrology, Department of Medicine, University of Texas Long School of Medicine, San Antonio, TX, United States of America; 2 Division of Nephrology, Penn State Hershey College of Medicine, Hershey, PA, United States of America; Ehime University Graduate School of Medicine, JAPAN

## Abstract

Interleukin-10 (IL-10), a cytokine with anti-inflammatory effects, is produced by renal parenchymal cells and bone marrow derived cells. Both endogenous and exogenous IL-10 are protective in cisplatin-induced acute kidney injury. However, the source of endogenous IL-10 in cisplatin-induced nephrotoxicity is not clear. Bone marrow chimera experiments in IL10-KO mice indicated that bone marrow derived cells were the primary source of IL-10 in cisplatin nephrotoxicity. Cell specific deletion of IL-10 in T regulatory cells and dendritic cells was accomplished using Foxp3 and CD11c driven cre recombination in *IL10*^*flox/flox*^ mice, respectively. Upon treatment with cisplatin, both the *IL10*^*flox/flox*^ and the Foxp3^YFP-Cre^ x *IL10*^*flox/flox*^ mice developed similar degrees of kidney injury. However, mice with the dendritic cell deletion of IL-10 showed more severe structural and functional changes in the kidney compared to the *IL10*^*flox/flox*^ mice. These results indicate that IL-10 from dendritic cells but not from T regulatory cells offers significant endogenous protection against cisplatin induced nephrotoxicity.

## Introduction

Cisplatin is an effective chemotherapeutic agent used for treatment of various solid tumors. Acute kidney injury is a major, and sometimes limiting, toxicity of cisplatin [1]. A large body of evidence indicates the involvement of inflammatory mechanisms in cisplatin-induced nephrotoxicity [2–7]. Various mediators of inflammation including chemokines, cytokines, TLRs and damage associated molecular patterns produced by renal parenchymal cells in response to cisplatin-induced injury [1–7]. These proinflammatory mediators recruit and activate leukocytes from the circulation and further aggravate kidney injury. In addition to proinflammatory molecules, anti-inflammatory pathways are activated in response to injury which inhibit ongoing cell injury and/or facilitate repair after the initial tubular injury [8–10] Previous reports suggest that some cytokines such as IL-4, IL-10, IL-3 and TGFβ may protect against tissue injury but the source of these cytokines and their role in renal injury/repair are poorly defined [11–13].

IL- 10 is an anti-inflammatory cytokine produced by a variety of cells; mainly Th2 cells, dendritic cells, T regulatory (Tregs) cells (CD4^+^CD25^+^Foxp3^+^), macrophages and renal proximal tubule cells [10, 14–16]. IL-10 attenuates the production of proinflammatory cytokines and chemokines [16, 17]. In addition, IL-10 inhibits the activation of immune cells and ameliorates renal injury in several models of kidney disease including lupus nephritis, ischemia reperfusion injury and transplantation [18–21]. We reported that cisplatin enhances renal IL-10 signaling and that endogenous production of IL-10 reduced cisplatin-induced acute kidney injury [9]. However, the source of IL-10 in cisplatin-induced nephrotoxicity is not clear. Among bone marrow derived cells, both Tregs and dendritic cells have been reported to reduce cisplatin nephrotoxicity [8, 10]. Both cells are also known to produce IL-10 [10, 14, 22], suggesting the possibility that IL-10 production by either or both of these cells is an endogenous mechanism to mitigate cisplatin-induced kidney injury.

In this study, we examined the cellular sources of IL-10 and their functional relevance during cisplatin-induced acute kidney injury. First, we performed bone marrow chimera experiments in IL10-KO mice to determine the role of renal parenchymal cells vs bone marrow derived cells in cisplatin-induced nephrotoxicity. Next, to analyze the role of endogenous IL-10 produced by Tregs and dendritic cells, we generated Foxp3^YFP-Cre^ x *IL10*^*flox/flox*^ mice, in which IL-10 is selectively inactivated in Foxp3^+^Treg cells, and *CD11c-Cre* x *IL10*^*flox/flox*^ mice, in which IL-10 is selectively deleted in CD11c^+^ dendritic cells.

## Methods

### Mice

All the experiments were performed using 8–10 week old C57BL/6, IL10 knockout, *IL10*^*flox/flox*^*; IL10*^*flox/flox*^*CD11c-Cre* and *IL10*^*flox/flox*^ Foxp3^YFP-Cre^ mice. All mice were on a C57BL/6 background. All procedures were approved by the Institutional Animal Care and Use Committee at University Texas Health Sciences, San Antonio (protocol 20160044AR)

### Cisplatin induced acute kidney injury

Acute kidney injury was induced in mice by a single intraperitoneal injection of cisplatin (20 mg/kg body weight). Renal function was determined by measuring BUN and serum creatinine prior to the cisplatin injection and at 24h, 48h and 72h after injection. We used *IL10*^*flox/flox*^ injected with cisplatin as a control group and compared with *IL10*^*flox/flox*^*CD11c-Cre* and *IL10*^*flox/flox*^ Foxp3^YFP-Cre^ mice injected with cisplatin. Based on previous studies, we included at least 5 mice in each group for cisplatin induced acute kidney injury experiments. Because of the variability of the litter size, we used different numbers of mice for each experiment. We performed concurrent controls within each experiment and only compare the experimental groups with the corresponding control group. After 72h of cisplatin injection mice were sacrificed by isoflurane anesthesia followed by cervical dislocation.

### Chimeric mice

Chimeric mice were created using C57BL/6 mice and mice with a global deletion of IL-10 as either bone marrow donors or recipients. Donor mice were killed with sodium pentobarbital and the femurs removed and flushed with RPMI medium containing 10% fetal calf serum to isolate bone marrow cells. Unfractionated bone marrow cells were washed and re-suspended in PBS at a concentration of 20 million cells/ml. Recipient C57BL/6 mice and IL-10 knockout mice were lethally irradiated using Gammacell irradiator (two doses of 600 rads, 4h apart). 8h after irradiation, 10 million-donor bone marrow cells were injected into the lateral tail vein of recipients. Three sets of chimeric mice were created: WT-WT (wild type donor and recipients; a control for the transplantation procedure) WT-KO (wild type donor and IL-10 knockout recipient; these mice express IL-10 only in the cells of bone marrow origin); KO-WT (IL10 knockout donor and wild type recipient, these mice express IL-10 in all tissue except cells of bone marrow origin). Mice were maintained in specific pathogen free conditions and were used around 8 weeks after the bone marrow transplantation. We used WT-WT chimeric mice as a control group.

### Histology

Kidneys were fixed in 10% neutral-buffered formalin overnight, dehydrated, and embedded in paraffin. Tubular injury was assessed in Periodic acid-Schiff (PAS)-stained sections. The number of injured tubules (cast formation, sloughing of epithelial cells, apoptotic nuclei) were counted blindly in ten randomly selected 40X fields for each kidney and expressed as a percentage of total tubule profiles.

### Western blotting

Kidneys homogenized in lysis buffer were separated on Nupage SDS-PAGE and then transferred to polyvinyllidene difluoride membranes. After blocking, membranes were incubated with rabbit anti-caspase 3 antibody (Cell Signaling) followed by HRP conjugated goat anti-rabbit antibody. Next, membranes were washed and proteins on the membrane were detected using enhanced chemiluminescence detection reagent.

### Validation of *CD11c-Cre* mediated *IL-10* deletion in dendritic cells

To confirm the deletion of the *IL10* gene in dendritic cells, IL-10 transcripts were examined in CD11c cells purified from the spleens of *IL10*^*flox/flox*^ and *IL10*^*flox/flox*^*CD11c-Cre* mice using the MagniSort CD11c positive selection kit (Thermo Fischer Scientific). Briefly, single cells suspensions were prepared from the spleen and labeled with biotinylated anti-mouse CD11c antibody. After washing, Magnisort positive selection beads were added to the pellet and incubated at room temperature. The bead-bound dendritic cells were separated with a magnet and used for qRT-PCR to measure the *IL10* transcript levels.

### Validation of *IL10*^*flox/flox*^ Foxp3^YFP-Cre^ mediated *IL10* deletion in Tregs

To test the deletion of *IL10* in T reg cells, we prepared single cell suspensions from spleens of *Il10*^*flox/flox*^ and *IL10*^*flox/flox*^ *Foxp3*^*YFP-Cre x*^ mice and isolated CD4^+^CD25^+^ Treg cells using the Dynabeads FlowComp kit (Invitrogen Life Technologies). This method uses negative selection of CD4+ T cells, followed by positive selection of CD25+ regulatory T cells. First, an antibody mixture was added to the spleen single cell suspension to bind the non CD4+ T cells. Mouse Depletion Dynabeads were then added and the bound non CD4+ cells were removed with a magnet. The remaining untouched mouse CD4+ T-cells were then labeled with CD25 antibody and positively isolated using Dynabeads. The T reg cells adherent to Dynabeads were separated with a magnet and used for qRT-PCR to measure the *IL10* transcript levels.

### Statistical analyses

Statistical differences among groups were analyzed using *Student*’s t-test (two groups), and one-way ANOVA with Tukey's post hoc analysis; *p* < 0.05 was considered statistically significant. All data are expressed as mean ± SEM.

## Results

### IL-10 from bone marrow derived cells plays an important role in protection against cisplatin-induced nephrotoxicity

**Fig 1:**
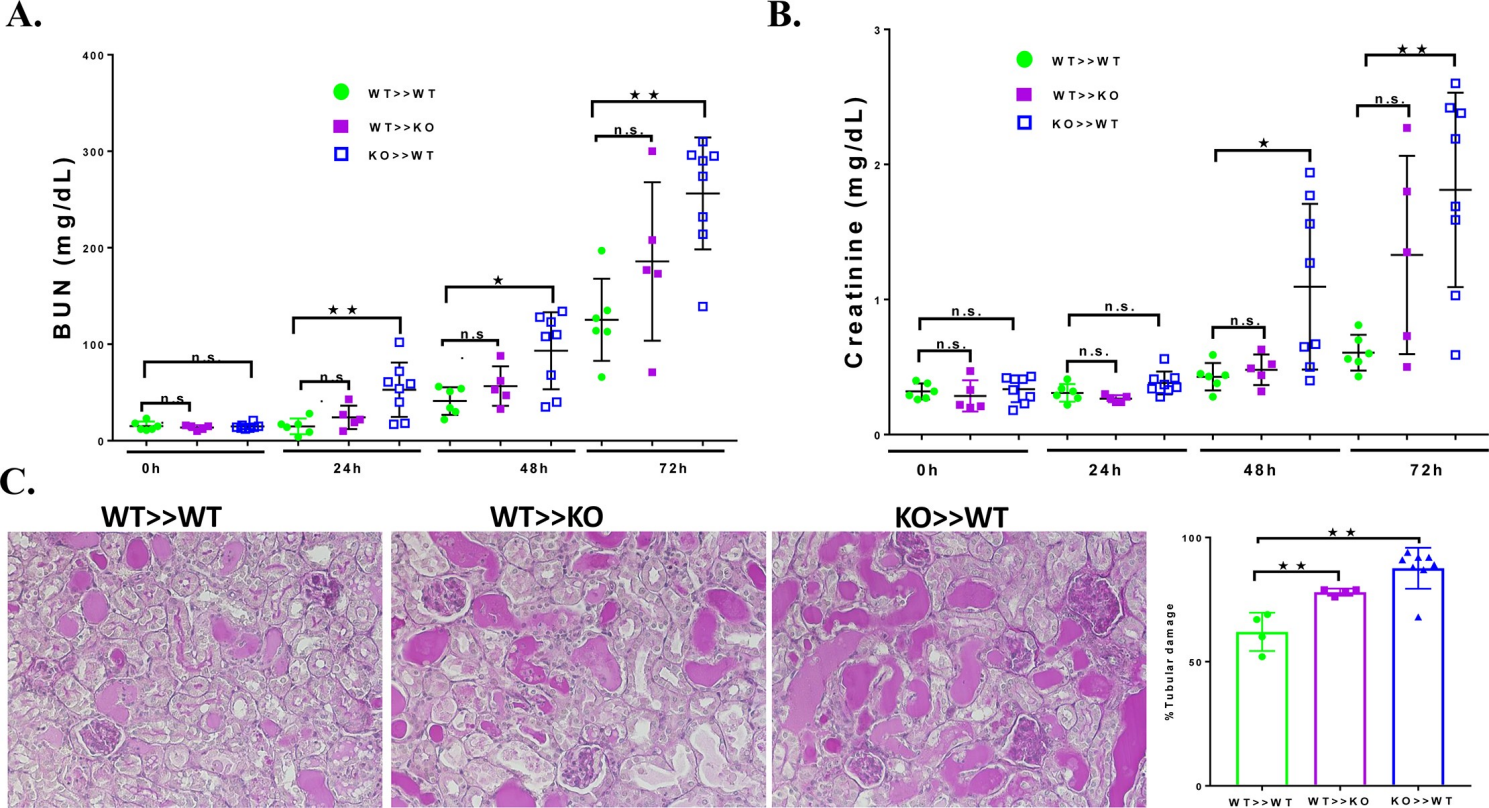
Cisplatin effect on kidney function and structure in chimeric mice. BUN (A) and creatinine (B) were measured before and at 24-hour intervals after injection of cisplatin in WT>>WT (n = 6), WT>>KO (n = 5) and KO>>WT (n = 8). C. PAS-stained sections of kidney 72 hours after cisplatin injection. D. Summary of the tubular damage score for each group of chimeric mice. Statistical analysis: WT>>WT was compared with WT>>KO and KO>>WT for each time point using one-way ANOVA with Tukey's post hoc analysis; Mean ± SEM. *p < 0.05, **p < 0.01, ns, not-significant.

To investigate the source of IL-10 which mediates protection in cisplatin toxicity, we performed bone marrow transplantation to create three sets of chimeric mice; WT to WT; WT- to KO; and KO to WT and tested the differential role of bone marrow cell-derived versus parenchyma-derived IL-10 in cisplatin induced nephrotoxicity. We previously reported that bone marrow transplantation results in a high degree of chimerism and does not alter the distribution of circulating leukocyte subtypes and the ability of circulating immune cells to produce TNFα [5]. Bone marrow chimeric mice were treated with cisplatin (20 mg/kg/body weight) or saline and evaluated for renal dysfunction by measuring serum creatinine and BUN. WT-WT chimeric mice developed significant renal failure at 48 and 72h **(Fig 1A&1B)**. The serum creatinine and BUN values at 72h are similar to what we observed previously in non-transplanted WT mice [3, 4, 8, 9, 23, 24]. These results indicate that bone marrow transplantation does not alter the susceptibility to cisplatin nephrotoxicity. Similarly, WT-KO mice also exhibited increasing BUN and serum creatinine values at 48h and 72h **(Fig 1A&1B)**. Although the BUN and creatinine values were numerically greater than in WT-WT mice, the difference was not statistically significant. However, KO-WT mice had a significantly greater increase in the BUN and serum creatinine values at 72h compared to the other groups. This result indicates that IL10 from bone marrow derived cells is important in ameliorating cisplatin kidney injury. The functional changes in the chimeric mice were also reflected in structural changes in kidney. At 72h after cisplatin treatment, kidneys with a wild type background had obvious tubular injury as evidenced by cast formation, loss of brush border membrane, sloughing of tubular epithelial cells, and dilation of tubules. These changes were more pronounced in the KO-WT mice (**Fig 1C)**.

### T regulatory cell-derived IL-10 does not significantly reduce cisplatin-induced nephrotoxicity

**Fig 2:**
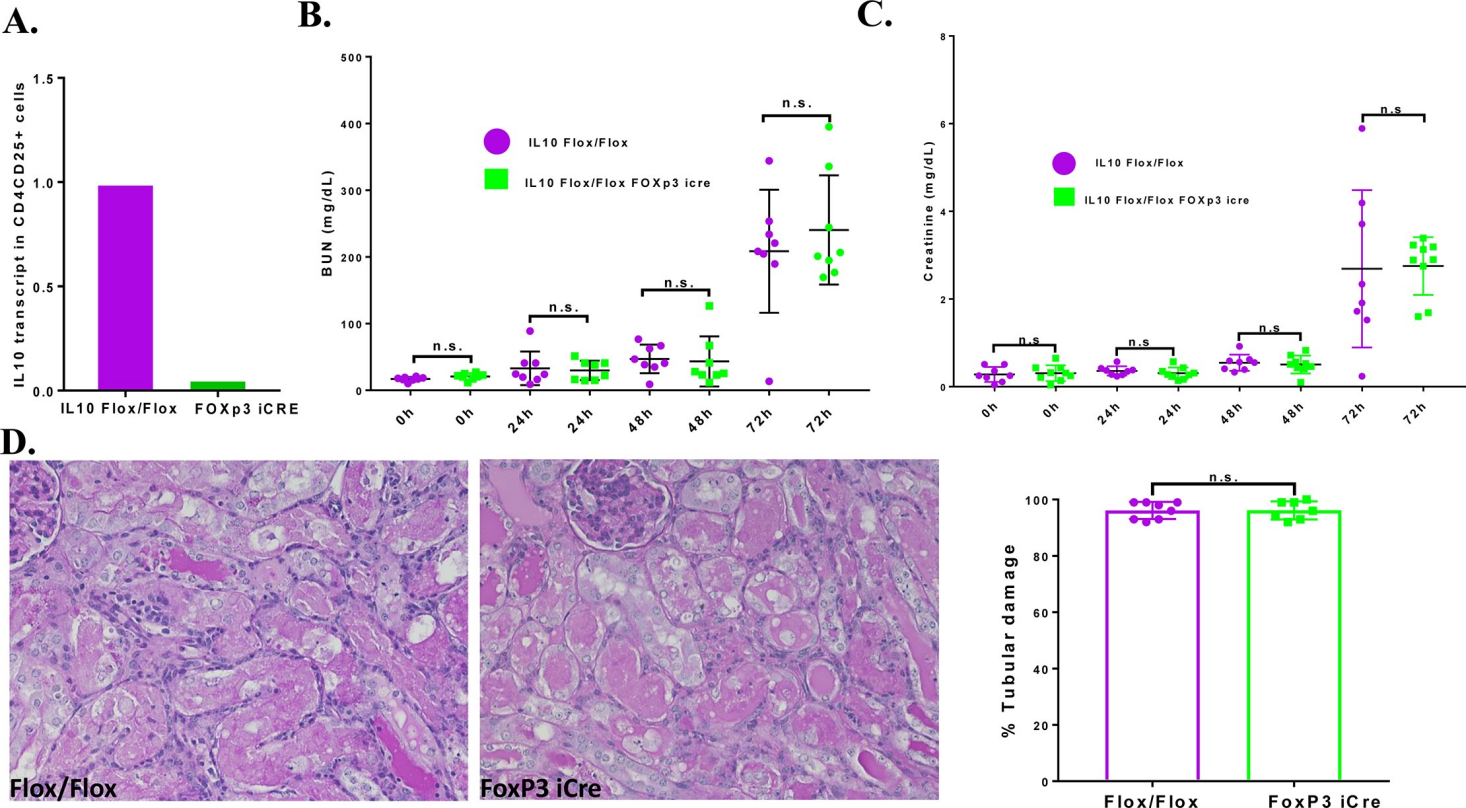
Effect of IL-10 depletion in Treg cells on cisplatin nephrotoxicity: A. Validation of *Foxp3*^*icre*^ mediated *IL-10* deletion in Treg cells. A. CD4/CD25+ cells isolated from spleens of both *IL10*^*flox/flox*^ and *IL10*^*flox/flox*^ *Foxp3*^*icre*^ mice and IL-10 transcript levels measured by qRT-PCR. B. BUN and creatinine levels in *IL10*^*flox/flox*^ (n = 8) and *Il10*^*flox/flox*^ *Foxp3*^*icre*^ *(n = 9)* mice injected with cisplatin. C. Histology of kidneys 72 hours after cisplatin injection. D. Summary of the tubular damage score for each group. Statistical analysis: Student’s unpaired t test; Mean ± SEM. *p < 0.05, ns, not-significant.

Since the chimera results indicated that bone marrow derived cells were important sources of IL-10 during cisplatin nephrotoxicity, we explored the role of Treg cells by producing a Treg specific IL-10 knockout. First, we validated the deletion of *IL10* in CD4^+^CD25^+^ cells by qRT-PCR. The results showed that *IL-10* transcript levels in CD4^+^CD25^+^ cells from *Il10*^*flox/flox*^ Foxp3^YFP-Cre^ mice were reduced by 87% compared to the cells from *IL10*^*flox/flox*^ mice **(Fig 2A)**. These results indicate effective iCre recombination in CD4^+^CD25^+^ Treg cells. *IL10*^*flox/flox*^ and *IL10*^*flox/flox*^ mice *Foxp3*^*YFP-Cre*^ were treated with cisplatin and renal function was assessed by measuring the levels of BUN and serum creatinine. Both the *IL10*^*flox/flox*^ mice and the *IL10*^*flox/flox*^ *Foxp3*^*YFP-Cre*^ mice treated with cisplatin showed minimal increases in the levels of BUN and serum creatinine at 24h; with progressive increases at 48h and 72h **(Fig 2B&2C).** There was no significant difference in the serum creatinine and BUN levels between the two genotypes. *IL10*^*flox/flox*^ and *Foxp3*^*YFP-Cre*^ mice treated with saline also exhibited comparable levels of BUN and serum creatinine. Structurally, *IL10*^*flox/flox*^ mice sacrificed 72h after cisplatin treatment showed severe tubular injury as characterized by cast formation, loss of brush border membranes, sloughing of epithelial cells, and dilation of tubules (**Fig 2D**). *IL10*^*flox/flox*^ *Foxp3*^*YFP-Cre*^ mice showed similar structural changes as the *IL10*^*flox/flox*^ mice. Semi quantification of tubule damage in both groups showed no significance difference. These results suggest that CD4^+^CD25^+^ Treg cells are not a major source of IL-10 in offering protection against cisplatin-induced nephrotoxicity.

### Dendritic cell derived IL-10 plays a significant role in protection against cisplatin-induced nephrotoxicity

**Fig 3:**
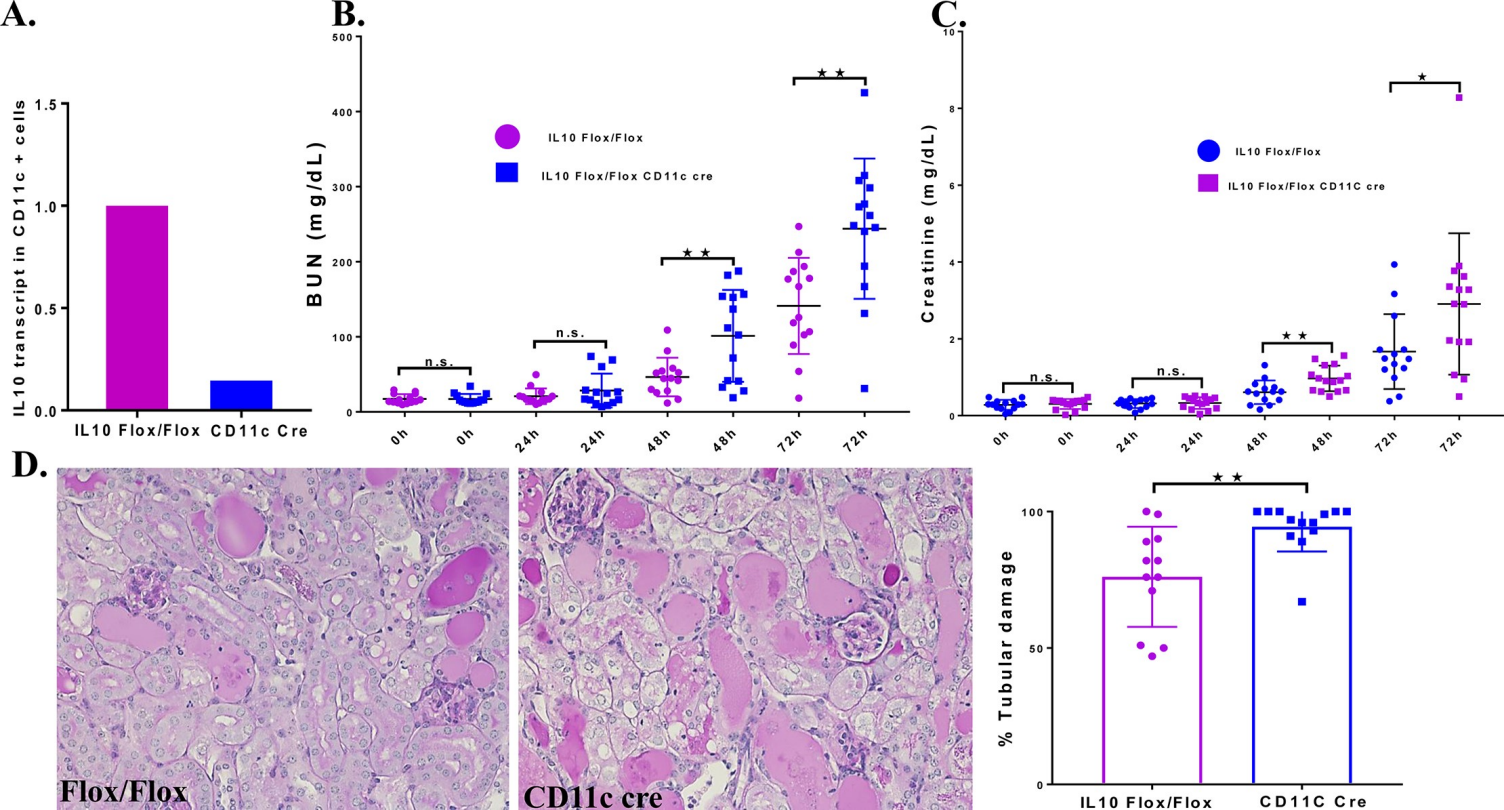
Effect of IL-10 depletion in dendritic cells on cisplatin nephrotoxicity. : A. Validation of CD11c-Cre mediated Il-10 deletion in dendritic cells. CD11c + cells were isolated from spleens of both Il10flox/flox and CD11cCre x*Il10*^*flox/flox*^ and Il-10 transcript levels measured by qRT-PCR. B. BUN and C. creatinine levels in *Il10*^*flox/flox*^ (n = 14) and *CD11c*^*Cre*^ x*Il10*^*flox/flox*^ (n = 14) mice injected with cisplatin. D. Histology of kidneys 72 hours after cisplatin injection. Summary of the tubular damage score for each group of mice. Statistical analysis: Student’s unpaired t test;. Mean ± SEM. *p < 0.05, **p < 0.01, ns, not-significant.

Previously, we reported that dendritic cells, which are the most abundant immune cells in the normal kidney, are protective against cisplatin-induced nephrotoxicity [8]. This conclusion was based on studies performed in *CD11c-DTRtg* mice in which the expression of the simian diphtheria toxin receptor driven by the CD11c promoter targets dendritic cells for DT mediated cell death. Based on these results, we hypothesized that IL-10 from dendritic cells might offer protection from cisplatin induced nephrotoxicity. To test this, we created *CD11c*^*Cre*^ x*IL10*^*flox/flox*^ mice and confirmed the deletion of *l10* in CD11c splenic cells by qRT-PCR. The results showed that CD11c splenic cells isolated from *CD11c*^*Cre*^ x*Il10*^*flox/flox*^ mice had >95% reduction in *Il10* transcript levels compared to cells from *Il10*^*flox/flox*^ mice **(Fig 3A)**. These results indicate effective CD11c cre recombination of *IL10* in dendritic cells. Analysis of renal function revealed that *Il10*^*flox/flox*^ mice treated with cisplatin showed minimal increases in the levels of BUN and serum creatinine at 24h, with large increases at 48h and 72h. Of note, *CD11c*^*Cre*^ x*Il10*^*flox/flox*^ mice showed significantly greater BUN and serum creatinine levels at 48h and 72h compared to *Il10*^*flox/flox*^ mice **(Fig 3B & 3C)**. Analysis of structural changes in the kidney 72h after the cisplatin injection showed more severe tubular damage in the *CD11c*^*Cre*^ x*Il10*^*flox/flox*^ kidneys compared to *Il10*^*flox/flox*^ mice **(Fig 3D)**. These results suggest that CD11c cells are an important source of IL-10 in offering protection against cisplatin-induced nephrotoxicity.

### IL-10 depletion in dendritic cells increases apoptosis, inflammatory gene expression and kidney injury markers

**Fig 4:**
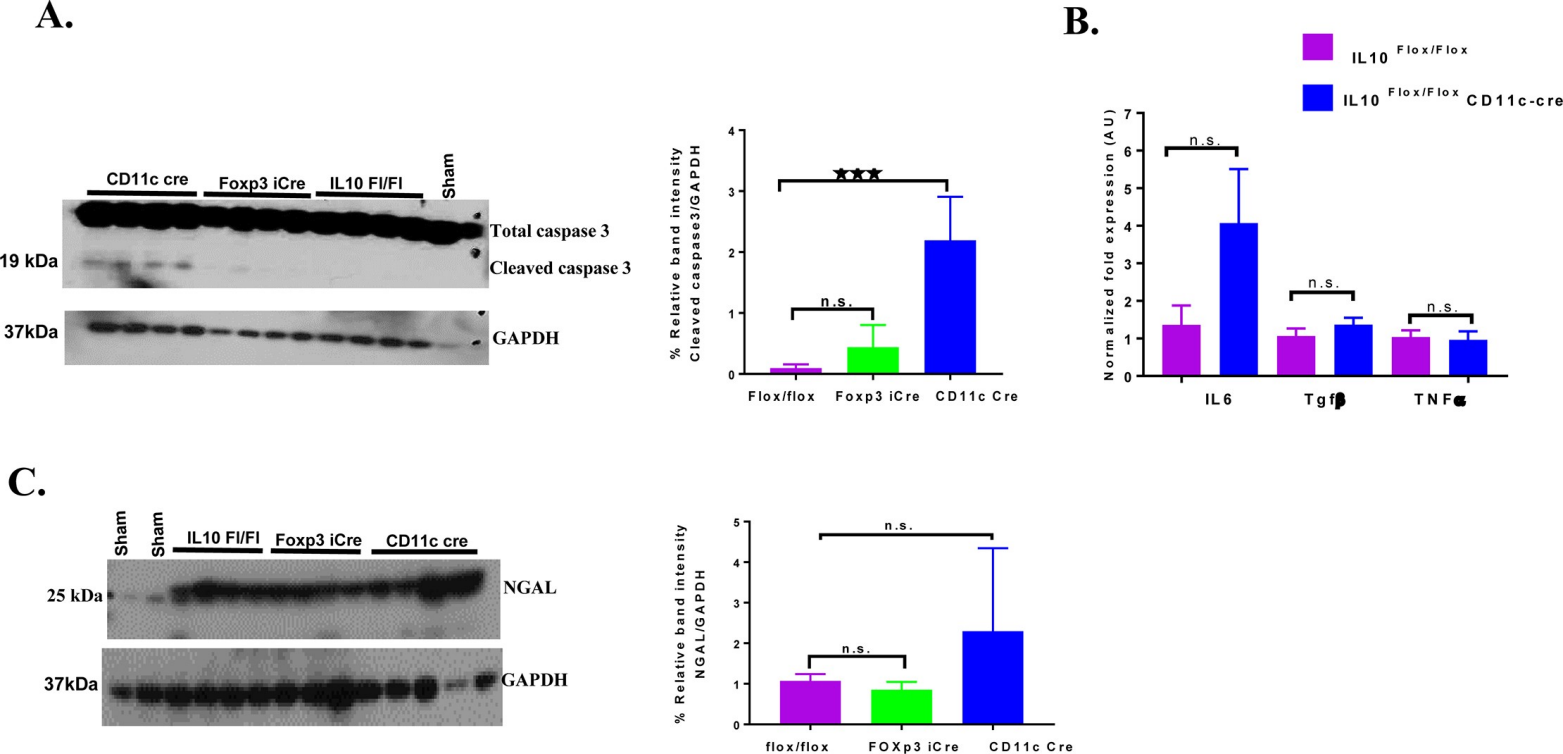
Increased apoptosis and proinflammatory gene expression in *CD11c*^*Cre*^ x*Il10*^*flox/flox*^ mice injected with cisplatin. A. Cisplatin-induced cleaved of caspase 3 levels measured by western blot. Cleaved capase 3 was measured in kidneys from *Il10*^*flox/flox*^, Foxp3^YFP-Cre^x *Il10*^*flox/flox*^ *CD11c*^*Cre*^ xIl10^flox*/flox*^ mice, 72 hr after cisplatin injection. B. Cytokine gene expression measured 72h after injection of cisplatin by qRT-PCR. The expression levels normalized to expression of GAPDH and expressed relative to *Il10*^*flox/flox*^ mice injected with cisplatin as a control. C. Kidney injury marker NGAL levels. NGAL was measured in kidneys from *Il10*^*flox/flox*^, Foxp3^YFP-Cre^x *Il10*^*flox/flox*^ *CD11c*^*Cre*^ xIl10^flox*/flox*^ mice 72 hr after cisplatin injection by Western blot. Statistical analysis: one-way ANOVA with Tukey's post hoc analysis comparing flox/flox mice with the other genotypes. Mean ± SEM. *p < 0.05, **p < 0.01, ns, not-significant.

Inflammation and tubular cell apoptosis are mechanisms underlying cisplatin induced AKI [3]. We found that deletion of IL-10 in dendritic cells resulted in a significant increase in cleaved caspase 3 in the kidney compared to Foxp3^YFP-Cre^x *Il10*^*flox/flox*^ and *Il10*^*flox/flox*^ mice **(Fig 4A)**. Cisplatin increases a number of proinflammatory cytokines in the kidney which contribute to renal injury [3]. IL-10 is known to inhibit the production of certain cytokines, chemokines and adhesion receptors [16, 17]. Therefore, we investigated the impact of deletion of Il-10 from dendritic cells on the expression of pro-inflammatory cytokines. Dendritic cell IL-10 knockout mice treated with cisplatin showed a trend towards increased expression of *Il6*, though not statistically significant, but similar expression of *tgfβ*, *and tnfα* in comparison with *Il10*^*flox/flox*^ mice treated with cisplatin **(Fig 4B)**. Finally, Dendritic cell IL-10 knockout mice treated with cisplatin showed a trend, though not statistically significant, towards increased levels of NGAL (Neutrophil gelatinase associated lipocalin) when compared with Foxp3^YFP-Cre^x *Il10*^*flox/flox*^ and *Il10*^*flox/flox*^ mice treated with cisplatin **(Fig 4C).**

## Discussion

In this mouse model of cisplatin induced nephrotoxicity, cisplatin increases IL-10 levels in plasma, renal IL-10 mRNA and IL-10R1 levels and STAT signaling, and this endogenous IL-10 offers protection against AKI [8, 9]. Thus, IL-10 deficient mice exhibit much more severe kidney injury than do wild type mice treated with cisplatin [9]. However, the source of IL-10 which accounts for this renal protection during acute kidney injury is not known. In this study, we demonstrate that IL-10 from bone marrow derived cells is critical for protection against cisplatin-induced nephrotoxicity. More specifically, Il-10 from dendritic cells plays a significant protective role against cisplatin-induced nephrotoxicity, whereas IL-10 from Treg cells is not a major factor. Further, deletion of IL-10 production by dendritic cells is associated with increased cleaved caspase 3 and proinflammatory cytokines.

IL-10 is produced by a broad range of tissues and cells, including immune cells, and glomerular and tubular epithelial cells [14, 15]. Cisplatin induced nephrotoxicity is associated with the influx of bone marrow derived inflammatory cells [8, 9, 23]. To differentiate the contribution of bone marrow derived vs renal parenchymal derived IL10 production, we created bone marrow chimeric mice. These experiments revealed a significant role for bone marrow derived IL-10 in mitigating cisplatin induced nephrotoxicity. We previously used this approach to demonstrate the role of TNFα and TLR4 in cisplatin induced nephrotoxicity [5, 6]. In those studies, we found that TNFα and TLR4 expression by non-leukocytes was key to the development of nephrotoxicity whereas the current studies identified leukocytes as the important source of IL-10. A number of immune cells produce IL-10, including neutrophils, macrophages, B cells, dendritic cells and several T cell subsets, notably T reg cells [14, 22]. Based on the existing literature, we focused our investigation on Treg cells and dendritic cells. Treg cells suppress inflammation and reduce kidney injury in models of both acute and chronic kidney disease [25]. Using adoptive transfer in immune deficient mice, two groups have shown a protective role for Tregs in cisplatin nephrotoxicity [10, 26]. In the most recent study [26] the protection was shown to require TLR9 expression on the Treg cell. However, neither study determined the role of IL-10 production by Tregs in protection. Since Tregs cells are known to produce IL-10 and since both endogenous [9] and exogenous [18] IL-10 reduce cisplatin injury, we examined the possibility that IL-10 produced by Treg cells mediated protection against cisplatin nephrotoxicity. Interestingly, deletion of IL-10 in Treg cells, driven by a Foxp3-specific cre recombinase, did not affect either the functional or the histologic injury induced by cisplatin (Fig 3). We conclude that the protective actions of Treg cells noted by other groups are mediated through other pathways. As a corollary, we conclude that Treg cells are not a major source of endogenous IL-10, which mediates protection from cisplatin injury (Fig 1). In this regard, *Foxp3*^*YFP-Cre*^ *x Il10*^*flox/flox*^ mice still possess other IL-10^+^ regulatory immune cells. Dendritic cells are known to produce IL-10 and are the most abundant immune cells in the normal kidney. We had previously shown that CD11c+ dendritic cells (or mononuclear phagocytes) are protective in cisplatin nephrotoxicity [8] and that cisplatin increased IL-10 expression in kidney [9]. Using a cell ablation model, we provided evidence that IL-10 production by dendritic cells mediated a portion of the protection provided by dendritic cells [9]. In the current study, we used a genetic approach to assess the significance of IL-10 production by dendritic cells in cisplatin toxicity. IL-10 was deleted from dendritic cells using a DC-specific (CD11c) cre recombinase. Compared to mice with an intact IL-10 gene, mice with the dendritic cell deletion of IL-10 showed significantly worse kidney function and more severe histologic kidney damage (Fig 3). These results support a significant role for dendritic cell-derived Il-10 in cisplatin-induced nephrotoxicity. These results are in support of our previous studies performed in CD11c-DTRtg mice in which the expression of the simian diphtheria toxin receptor driven by the CD11c promoter was used to target dendritic cells for DT mediated cell death [8, 9]. We note that the distinction between kidney dendritic cells and macrophages is not clear cut as many cells exhibit overlapping expression of classical dendritic cell and macrophage markers such as CD11c, CD11b and F4/80 [8, 27, 28], prompting some investigators to refer to the cells as renal mononuclear macrophages [29]. Accordingly, although we use the term dendritic cell here, we acknowledge that the CD11c-cre may have also deleted IL-10 from cells which may have macrophage features. We also note that the deletion of IL-10 from dendritic cells, while increasing injury, did not result in as severe a phenotype as global IL-10 deletion, in which BUN and creatinine values are dramatically elevated by 24 hours after cisplatin injection [9]. Thus, there may be other sources of IL-10 production which are important in mitigating cisplatin kidney injury. Likewise, the deletion of IL-10 from dendritic cells did not result in as severe a phenotype as did deletion of dendritic cells themselves using the CD11c-DTR [8]. This finding would suggest that dendritic cells may be modulating injury though multiple pathways, some of which do not require IL-10.

In summary, we have demonstrated that the production of IL-10 by bone marrow derived immune cells is important for offering protection from cisplatin-induced nephrotoxicity. Specifically, IL-10 from dendritic cells, but not from Treg cells, plays a significant role in reducing cisplatin-induced nephrotoxicity. Further work is required to elucidate the targets of IL-10 in cisplatin nephrotoxicity and other potential sites of IL-10 production.

## Supporting information

S1 Checklist (PDF)

S1 File (DOCX)

S1 Raw images (DOCX)
