# Supplementary material for: IL-10 from dendritic cells but not from T regulatory cells protects against cisplatin-induced nephrotoxicity
Source: PLoS One. 2020 Sep 8;15(9):e0238816. doi: 10.1371/journal.pone.0238816 (PMC7478814; doi:10.1371/journal.pone.0238816)
Supplement: S1 File — (DOCX) [file pone.0238816.s002.docx]

**Supplementary files:**

**Fig.1: supporting information**

| WT>>WT  0h | WT>>KO  0h | KO>>WT  0h | WT>>WT  24h | WT>>KO  24h | KO>>WT  24h | WT>>WT  48h | WT>>KO  48h | KO>>WT  48h | WT>>WT  72h | WT>>KO  72h | KO>>WT  72h |
| --- | --- | --- | --- | --- | --- | --- | --- | --- | --- | --- | --- |
| 12  15  18  12  23  11 | 15  15  10  12  16 | 14  15  16  12  13  14  21  13 | 9  4  14  17  28  17 | 43  22  27  19  10 | 102  72  18  40  59  61  54  17 | 51  30  54  56  34  22 | 88  53  47  62  33 | 128  108  35  68  134  123  110  40 | 197  113  127  135  114  66 | 300  173  177  208  71 | 295  274  232  214  310  290  296  139 |

Fig.1 A. BUN measured before and at 24-hour intervals after injection of cisplatin in chimeric mice

| WT>>WT  0h | WT>>KO  0h | KO>>WT  0h | WT>>WT  24h | WT>>KO  24h | KO>>WT  24h | WT>>WT  48h | WT>>KO  48h | KO>>WT  48h | WT>>WT  72h | WT>>KO  72h | KO>>WT  72h |
| --- | --- | --- | --- | --- | --- | --- | --- | --- | --- | --- | --- |
| 0.32  0.29  0.38  0.26  0.4  0.27 | 0.22  0.2  0.21  0.33  0.47 | 0.28  0.42  0.41  0.23  0.43  0.41  0.18  0.33 | 0.41  0.29  0.22  0.32  0.34  0.27 | 0.28  0.24  0.27  0.3  0.24 | 0.35  0.37  0.28  0.42  0.34  0.56  0.41  0.33 | 0.45  0.38  0.44  0.43  0.59  0.28 | 0.52  0.32  0.44  0.63  0.49 | 0.65  0.67  0.5  1.27  1.94  1.56  1.77  0.4 | 0.7  0.6  0.54  0.56  0.81  0.43 | 1.35  0.73  2.27  1.8  0.5 | 2.42  2.19  1.59  1.03  2.38  1.69  2.6  0.59 |

Fig. 1B: Creatinine measured before and at 24-hour intervals after injection of cisplatin in chimeric mice

Fig. 1C: Summary of the tubular damage score for each group of chimeric mice.

| WT>>WT | WT>>KO | KO>>WT |
| --- | --- | --- |
| 69  60  67  52 | 79  79  76  78 | 89  92  87  91  94  92  88  68 |

Fig.2: Supporting information:

Fig. 2A: BUN levels in IL10^flox/flox^ and Il10^flox/flox^ Foxp3 icre mice injected with cisplatin

| flox/flox  0h | Foxp3 icre  0h | flox/flox  24h | Foxp3 icre  24h | flox/flox  48h | Foxp3 icre  48h | flox/flox  72h | Foxp3 icre  72 h |
| --- | --- | --- | --- | --- | --- | --- | --- |
| 17.04  20.93  10.22  18.5  18.01  16.1  19.21  15.58 | 11.68  19.96  20.93  27.26  24.82  17.13  21.81  22.85 | 88.79  24.53  40.89  40.89  23.95  16.18  19.77  8.99 | 14.6  15.19  22.2  37.97  51.4  40.74  16.18  40.74 | 46.73  9.02  62.31  40.58  66.81  38.6  76.7  35.05 | 11.89  22.95  41.4  23.36  25.41  126.47  27.94  67.05 | 220.66  13.5  204.57  189.51  253.37  233.64  344.1  208.16 | 206.65  169.26  176.53  200.93  194.7  395.07  244.27  335.6 |

Fig. 2B: Creatinine levels in IL10^flox/flox^ and Il10^flox/flox^ Foxp3 icre mice injected with cisplatin

| flox/flox  0h | Foxp3 icre  0h | flox/flox  24h | Foxp3 icre  24h | flox/flox  48h | Foxp3 icre  48h | flox/flox  72h | Foxp3 icre  72 h |
| --- | --- | --- | --- | --- | --- | --- | --- |
| 0.25  0.11  0.21  0.04  0.25  0.36  0.51  0.51 | 0.04  0.25  0.21  0.14  0.32  0.43  0.65  0.43  0.31 | 0.38  0.42  0.35  0.24  0.28  0.27  0.37  0.57 | 0.14  0.17  0.28  0.35  0.24  0.27  0.43  0.57  0.31 | 0.56  0.59  0.92  0.39  0.33  0.55  0.62  0.41 | 0.72  0.43  0.53  0.56  0.46  0.83  0.1  0.41  0.51 | 4.19  0.24  5.89  2.34  3.71  1.91  1.52  1.72 | 1.69  3.19  2.9  3.23  3.39  3.13  1.6  2.89  2.75 |

Fig. 2C: Histology of IL10^flox/flox^ and Il10^flox/flox^ Foxp3icre mice kidneys 72 hours after cisplatin injection

| Flox/flox | Foxp3 icre |
| --- | --- |
| 93  93  99  92  99  98  96  99 | 92  93  96  100  99  94  99 |

**Fig. 3: Supporting information:**

Fig. 3A: BUN levels in IL10 ^flox/flox^ and Il10 ^flox/flox^ CD11c cre mice injected with cisplatin

| flox/flox  0h | CD11c cre  0h | flox/flox  24h | CD11c cre  24h | Flox/flox  48h | CD11c cre  48h | Flox/flox  72h | CD11c cre  72 h |
| --- | --- | --- | --- | --- | --- | --- | --- |
| 27.2586  24.338  29.2056  24.338  14.0187  14.0187  16.3551  18.6916  11.8886  10.9805  13.2096  9.577  16.1818  12.707 | 24.338  34.0732  25.3115  21.4174  14.0187  11.6822  11.6822  13.3511  16.1818  13.0445  15.769  12.7142  15.769  11.4773 | 26.285  11.6822  35.0467  49.6495  14.4637  21.1393  17.134  10.0134  20.0853  16.806  15.9862  15.9862  16.3961  20.9051 | 69.1199  73.9875  60.3583  26.285  26.7023  28.9275  24.9221  17.134  8.608  15.9862  6.9684  12.2971  11.0674  16.3961 | 44.7819  11.6822  109.0343  54.5171  52.2919  30.0401  16.6889  81.2194  41.6002  44.4495  27.9234  58.1263  51.8578  25.6439 | 153.8162  182.0483  156.7368  111.9548  102.3587  152.4255  28.0374  136.8491  41.6002  41.0303  187.4858  33.0522  71.8031  18.8056 | 118.6197  53.918  125.8088  186.9159  246.996  106.8091  89.0076  176.9025  193.7543  166.97  102.5758  212.5598  177.798  18.2357 | 248.023  298.3465  276.7793  194.105  314.8643  240.3204  31.1526  308.1887  245.612  272.9656  425.1197  166.9706  261.5683  131.07 |

Fig. 3A: Creatinine levels in IL10 ^flox/flox^ and Il10 ^flox/flox^ CD11c cre mice injected with cisplatin

| flox/flox  0h | CD11c cre  0h | flox/flox  24h | CD11c cre  24h | Flox/flox  48h | CD11c cre  48h | Flox/flox  72h | CD11c cre  72 h |
| --- | --- | --- | --- | --- | --- | --- | --- |
| 0.3814  0.0424  0.2119  0.0847  0.4808  0.1923  0.2885  0.2885  0.3309  0.1838  0.3676  0.2941  0.4779  0.2941 | 0.0233  0.339  0.0847  0.3814  0.3846  0.3846  0.2115  0.3846  0.4044  0.2941  0.3309  0.1471  0.4779  0.4044  0.3 | 0.3205  0.2143  0.4233  0.4487  0.0763  0.3053  0.458  0.1527  0.2665  0.4103  0.2305  0.4103  0.3384  0.3384 | 0.5128  0.1923  0.4487  0.3846  0.4198  0.0382  0.3053  0.4962  0.1154  0.2308  0.1538  0.5  0.4615  0.3384  0.33 | 0.7377  0.4098  1.3115  0.8197  0.5725  0.2672  0.2672  0.4198  0.1586  0.626  0.6979  0.9855  0.626  0.626 | 0.9016  1.4754  1.3115  1.0656  0.8779  1.0305  0.4962  1.5649  0.626  0.9136  1.345  0.6619  0.6619  0.626  0.97 | 1.4754  0.4918  1.7213  3.9344  2.5954  1.7176  1.6031  3.1679  1.345  1.4888  0.9855  1.2012  1.2371  0.3743 | 3.2787  8.2787  3.7705  3.2787  2.9008  3.626  0.4962  3.3588  1.9202  1.9561  3.8973  1.9202  0.9495  1.0574  2.91 |
|  |  |  |  |  |  |  |  |

3C: Histology of IL10^flox/flox^ and Il10^flox/flox^ CD11c cre mice kidneys 72 hours after cisplatin injection

| Flox/flox | CD11c cre |
| --- | --- |
| 71  51  100  99  90  76  89  82  82  47  50  76 | 100  100  100  100  89  97  100  99  96  93  96  91  67 |
