# Supplementary material for: IL-10 from dendritic cells but not from T regulatory cells protects against cisplatin-induced nephrotoxicity
Source: PLoS One. 2020 Sep 8;15(9):e0238816. doi: 10.1371/journal.pone.0238816 (PMC7478814; doi:10.1371/journal.pone.0238816)
Supplement: S1 Raw images — (DOCX) [file pone.0238816.s003.docx]

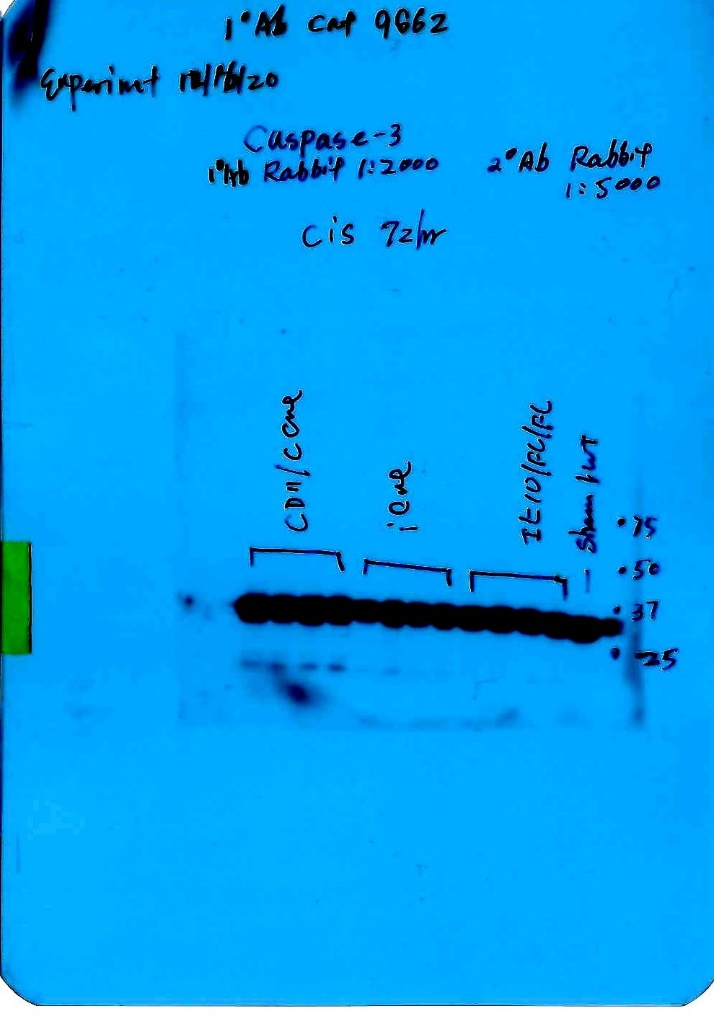
Figure 4A: Original western blot for caspase 3 and corresponding loading control (GAPDH) western blots


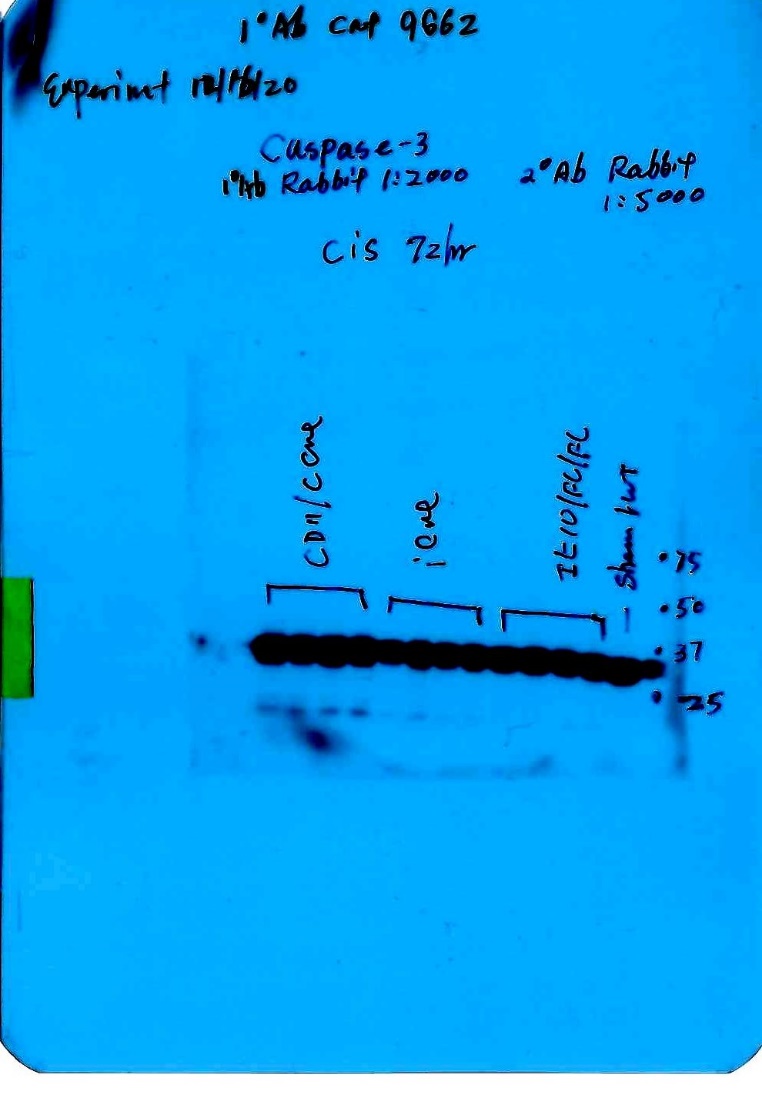

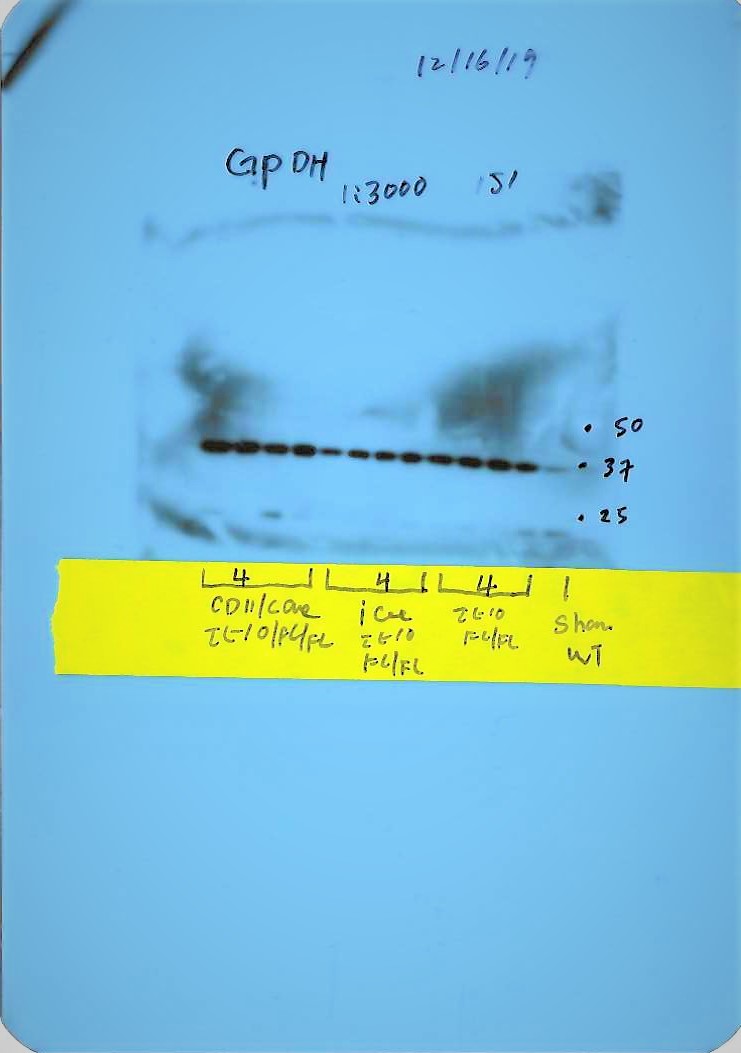


Fig. 4C. NGAL and corresponding loading control (GAPDH) western blot

NGAL Western Blot

GAPDH western blot for NGAL


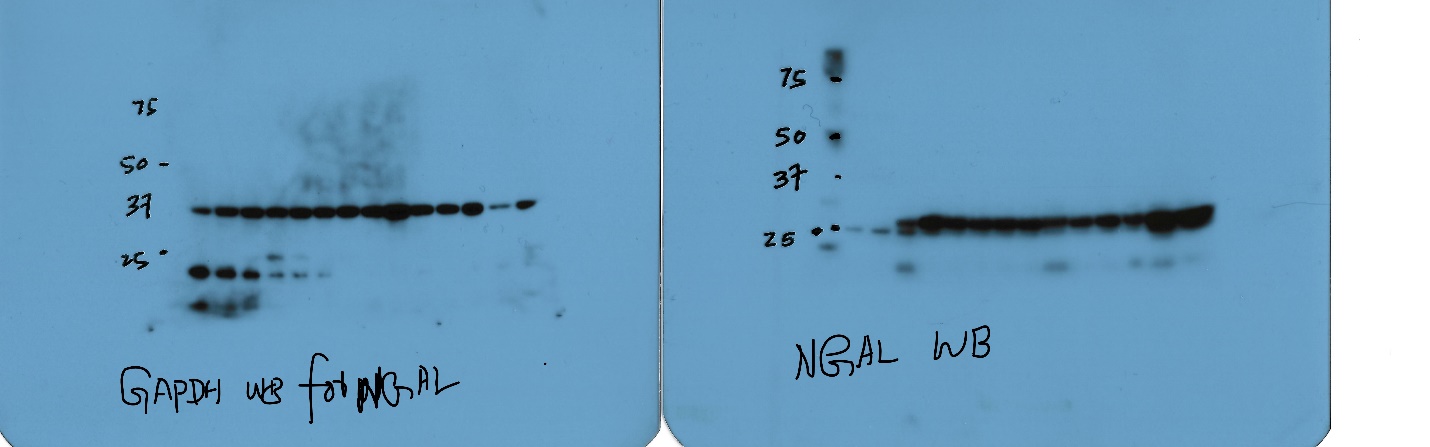


GAPDH

NGAL

**Foxp3 icre**

**CD11c cre**

**IL10 Fl/Fl**

**Sham**

**Sham**

**CD11c cre**

**Foxp3 icre**

**IL10 Fl/Fl**

**Sham**

**Sham**
